# Acupuncture for hyperlipidemia: Protocol for a systematic review and meta-analysis

**DOI:** 10.1097/MD.0000000000013041

**Published:** 2018-12-14

**Authors:** Qinyan Peng, Xiaobin Yao, Jiexiang Xiang, Yuanping Wang, Xinmei Lin

**Affiliations:** Guangzhou University of Chinese Medicine, Guangzhou, China.

**Keywords:** acupuncture, hyperlipidemia, protocol, systematic review

## Abstract

**Background:**

Hyperlipidemia is a major risk factor for cardiovascular and cerebrovascular diseases. Acupuncture has been widely applied in the treatment of hyperlipidemia. But its efficacy has not been evaluated scientifically and systematically. Therefore, we provide a protocol of systematic evaluation to assess the effectiveness and safety of acupuncture treatment on patient with hyperlipidemia.

**Methods:**

We will search the following databases electronically, including 3 English literature databases (i.e., PubMed, Embase, and Cochrane Library) and 4 Chinese literature databases (i.e., Chinese Biological and Medical database, China National Knowledge Infrastructure, VIP, and Wanfang Database). We will also search randomized-controlled trials about acupuncture treatment for hyperlipidemia and the search time limit is from its establishment to October 2018. The primary outcome is lipid-lowering efficacy. Secondary outcomes are total cholesterol, low-density lipoprotein cholesterol, triglyceride, and high-density lipoprotein cholesterol levels. We will use RevMan V.5.3 software as well to compute the data synthesis carefully when a meta-analysis is allowed.

**Results:**

This study will provide a high-quality synthesis to assess the effectiveness and safety of acupuncture treatment on patient with hyperlipidemia.

**Conclusion:**

The conclusion of our systematic review will provide evidence to judge whether acupuncture is an effective intervention for patient with hyperlipidemia.

## Abstract 2

Supplemental Digital Content is available in the text

## 1 Introduction

Hyperlipidemia refers to abnormalities in the quality and level of lipids in the blood, usually represented as elevated total cholesterol (TC) and/or triglyceride (TG), as well as a decrease in high-density lipoprotein cholesterol (HDL-C).^[1]^ Hyperlipidemia is a major risk factor for cardiovascular and cerebrovascular diseases. Hyperlipidemia could induce atherosclerosis, leading to coronary heart disease, stroke, and myocardial infarction, increasing the incidence and mortality rate of cardio-cerebrovascular diseases.^[2,3]^ In the United States, roughly 53% of adults, or more than 100 million, have elevated low-density lipoprotein cholesterol (LDL-C) levels.^[4]^ In addition, studies have shown that the overall prevalence of dyslipidemia among Chinese adults is as high as 40.40%, a significant increase from 2002.^[5]^ Elevated serum cholesterol levels in the population will lead to an increase of 9.2 million cardiovascular events in China between 2010 and 3030.^[5]^ The incidence of hyperlipidemia is gradually increasing, and it may deteriorate as the population ages. Therefore, prevention and control of hyperlipidemia is of great significance.^[6]^ Chinese and international research and clinical practice have proved that dyslipidemia can be prevented and controlled. Reducing cholesterol levels in the population can significantly reduce myocardial infarction, ischemic stroke events, and cardiovascular death, improve the quality of life of patients with cardiovascular disease, and effectively reduce their burden brought by the disease.^[7]^

Diet and lifestyle have a significant effect on dyslipidemia, and diet therapy and lifestyle improvement are the basic measures for the treatment of dyslipidemia.^[6]^ Whether or not lipid-lowering drugs are taken, it is essential to control diet and improve lifestyles.^[8]^ In terms of medical treatment, clinically used lipid-lowering drugs include statins, fibrates, niacin, resins, intestinal cholesterol absorption inhibitors, probucol, and other compound preparations.^[9]^ However, almost all major lipid-lowering drugs cause side effects. For example, statins may cause myalgia and other muscle symptoms in approximately 10% of patients treated, and an estimated 20% of patients have statin resistance or intolerance, so new treatments need to be explored.^[10]^

Acupuncture has been used to treat diseases for more than 2500 years and plays an important role in traditional Chinese medicine.^[11]^ This is a minimally invasive surgery in which a fine metal needle is inserted into a specific body point (acupoint) and slowly twisted manually to stimulate for therapeutic purposes. With simple operation, low cost, and few adverse reactions, acupuncture therapy is very popular among Chinese people. ^[12]^ In some western countries, patients and their families are increasingly demanding acupuncture as an adjuvant therapy.^[12]^ In recent decades, acupuncture has been widely used in the adjuvant treatment of patients with hyperlipidemia.^[13,14]^ A large number of clinical studies in China have shown that acupuncture can lower TC, TG, LDL-C and increase HDL-C in patients with dyslipidemia.^[13–15]^

However, due to the uneven quality of research, the efficacy of acupuncture for hyperlipidemia has not been accepted by the mainstream medical community, and there is no systematic review about the acupuncture treatment of hyperlipidemia. Based on this, this study intends to use evidence-based medicine evidence, search global clinical research on acupuncture treatment of hyperlipidemia, systematically evaluate the efficacy of acupuncture treatment of hyperlipidemia, and provide more scientific evidence for clinical treatment of hyperlipidemia.

## 2 Methods

### 2.1 Inclusion criteria for study selection

#### 2.1.1 Types of studies

All clinical randomized controlled trials (RCTs) of acupuncture for hyperlipidemia will be included in the review.

#### 2.1.2 Types of patients

The cases included in the trial are all patients with hyperlipidemia, not limited by age and race.

#### 2.1.3 Types of interventions

The treatment group adopts acupuncture therapy (without limitation on needle material, treatment point selection, operation method, needle retention time, and treatment course), while the control group is treated with internationally recognized therapy (such as oral statin therapy) or sham acupuncture.

#### 2.1.4 Types of outcome measures

##### 2.1.4.1 Primary outcomes

The primary outcome is lipid-lowering efficacy.

##### 2.1.4.2 Secondary outcomes

- TC
- LDL-C
- TG
- HDL-C

### 2.2 Search methods for the identification of studies

#### 2.2.1 Electronic searches

We will search the following databases electronically, including 3 English literature databases (i.e., PubMed, Embase, and Cochrane Library) and 4 Chinese literature databases (i.e., Chinese Biological and Medical database, China National Knowledge Infrastructure, VIP, and Wanfang database). We will also search RCTs about acupuncture treatment for hyperlipidemia and the search time limit is from its establishment to October 2018. Besides, we will retrieve unpublished protocols and summary results by searching the clinical trial registry at https://clinicaltrials.gov/mesh. The search uses a combination of subject words and free words, and the search strategy is determined after multiple presearches. The search terms include acupuncture, hyperlipidemia, and randomization. Meanwhile, we will search the literature included in the research reference and original literature which are subject-related and included by systematic reviews, so as to supplement and obtain relevant literature and ensure the recall ratio. The search strategy of PubMed will be shown in supplemental digital content (Appendix A).

#### 2.2.2 Searching other resources

We will also have manual retrieval for relevant conference reports, and contact experts in the field and corresponding authors to obtain important information that cannot be obtained by the above retrieval.

### 2.3 Data collection and analysis

#### 2.3.1 Selection of studies

**Figure 1:**
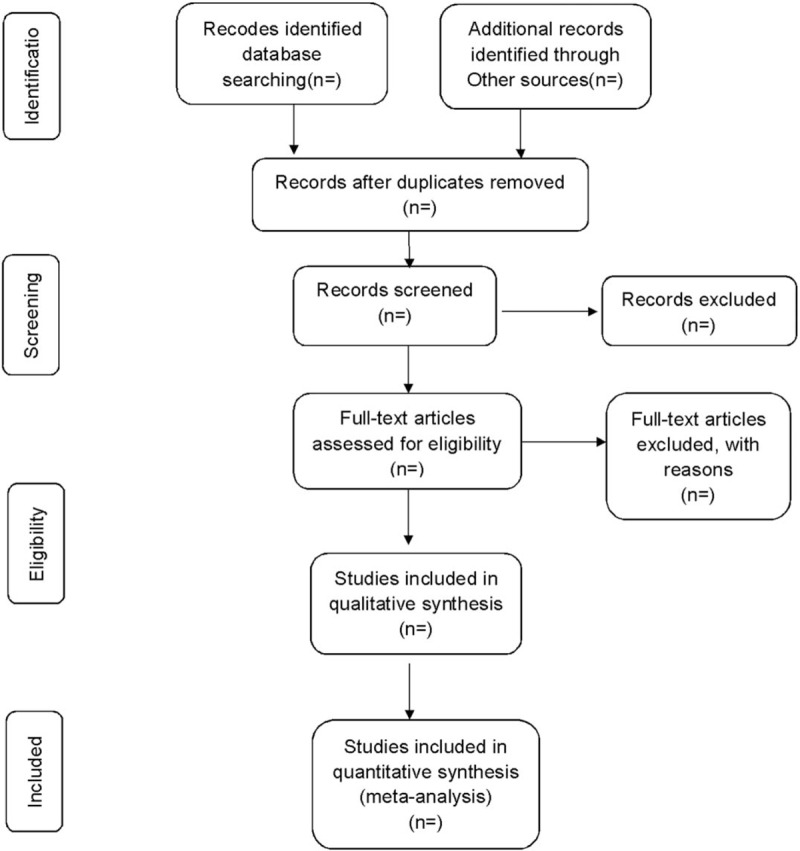
Flow diagram of study selection process.

Researchers will discuss and determine the screening criteria within the group before searching the studies. The corresponding research members will import the retrieved studies into the document management system of EndnoteX7 for repetition removal. We will then exclude the apparently unqualified literature by reading the headings and abstracts, and determine the final included literature by reading the full text, discussing within the group and contacting the author to know more about the research details. The final list of included studies will be converted to the format of Microsoft Excel. Both the information retrieval and the literature screening will be independently operated by 2 research members. Finally, another research member will resolve the inconsistency and check the final included studies. Study selection is summarized in a PRISMA flow diagram (Fig. 1).

#### 2.3.2 Data collection and management

Data collection will be conducted by 2 researchers using EpiData 3.1 software for double entry, and finally another research member will detect data consistency and check the final database. Data for collection include disease diagnosis, tools for diagnosing or screening hyperlipidemia, combined disease, course of disease, stage, disease severity, sample size, age, interventions and details about the control group, follow-up, outcomes, findings, and adverse event details. Any disagreement on data collection will be resolved through discussions or negotiations with the third arbitrator. If the data provided in the study are unclear, missing, or presented in a form that is not extractable or difficult to extract reliably, we will contact the author of the study for clarification.

#### 2.3.3 Assessment of risk of bias in included studies

According to the Cochrane Collaboration's tool for assessing risk of bias provided by Cochrane Handbook for Systematic Reviews of Interventions, we will assess from 7 dimensions: random sequence generation, allocation concealment, blinding of patients, blinding of testers, blinding of outcome evaluators, outcome data incompletion, and selective reporting of 7 dimensions for evaluation. The results of the assessment will be divided into 3 levels: low risk, unclear, and high risk. The assessment will be conducted independently by 2 trained research members, and the inconsistencies will be resolved through intragroup discussions, contacting authors to determine details with the third-party arbitrator.

#### 2.3.4 Measures of treatment effect

The enumeration data are expressed as relative risk, the measurement data adopt mean difference, and each effect amount is expressed in 95% confidence interval.

#### 2.3.5 Dealing with missing data

For studies in which the data are missing, the researcher will try to obtain information by contacting the corresponding author of the study. If contact is lost, we will build our analysis on the available data.

#### 2.3.6 Assessment of heterogeneity

Prior to statistical analysis, the chi-squared test will be used to determine the homogeneity of the study. If the resulting *P* value exceeds .1, it indicates significant heterogeneity of the test. The cause of the heterogeneity will be analyzed and a subgroup analysis will be performed.

#### 2.3.7 Assessment of reporting bias

When more than 10 studies are included, the symmetry of the funnel plot will be first used to determine whether there is a publication bias. If the image is unclear, Egger test will be performed for quantitative analysis using STATA 12.0 software.

#### 2.3.8 Data synthesis

Meta-analysis will be performed using RevMan 5.3 software. When there is no statistical heterogeneity among the results, a fixed-effects model will be used for meta-analysis. When there is statistical heterogeneity among the results, the heterogeneity source will be further analyzed and a random-effects model will be used for meta-analysis after excluding the effects of significant clinical heterogeneity. When there is significant clinical heterogeneity, we will use subgroup analysis or sensitivity analysis, or only descriptive analysis.

#### 2.3.9 Subgroup analysis

The following subgroup analysis will be performed to assess the heterogeneity of the research

Clinical consideration

- Different acupoints
- Different courses of treatment
- Different races

Methodology consideration

- Tests with unclear or high risks of bias

#### 2.3.10 Sensitivity analysis

Sensitivity analysis is an important method primarily used to assess the robustness and reliability of the combined results of meta-analysis. It is a commonly used sensitivity analysis method to combine the effect size after eliminating each of the included studies, or after changing the inclusion or exclusion criteria or eliminating certain types of studies. For possible low-quality studies, sensitivity analysis is required.

#### 2.3.11 Ethics and dissemination

This systematic review and meta-analysis will not require ethical approval because there are no data used in our study that are linked to individual patient data. In addition, findings will be disseminated through conference presentations and peer-review publications.

## 3 Discussion

Dyslipidemia characterized by LDL-C or elevated TC is an important risk factor for atherosclerotic cardiovascular disease (ASCVD).^[16]^ Lowering the LDL-C level can significantly reduce the risk of morbidity and mortality from ASCVD.^[17]^ Acupuncture has been used in China for treating diseases for thousands of years. In recent decades, studies have found that acupuncture treatment has a good effect on patients with hyperlipidemia. However, the specific mechanism is not clear, and its clinical efficacy has not been recognized by international authoritative medical organizations, it is therefore necessary to systematically assess the safety and efficacy of acupuncture in treating patients with hyperlipidemia. Since there is no systematic review about dyslipidemia for acupuncture, we hope that this systematic review will help clinicians make decisions in practice and promote the progress of acupuncture research.

However, there are some potential limitations to this study. For example, different acupoints and difference in the level of methodological quality included in the trials may result in significant heterogeneity. In addition, limited to language ability, we only search for documents in English and Chinese, and may ignore studies or reports in other languages.

## Author contributions

Xinmei Lin is the guarantor of the article. The manuscript was drafted by Xinmei Lin and Xiaobin Yao. Jiexiang Xiang and Xinmei Lin developed the search strategy. Yuanping Wang and Xiaobin Yao were independently screened the potential studies and extracted data. Xinmei Lin and Xiaobin Yao were assessed the risk of bias and finished data synthesis. Xinmei Lin was arbitrated any disagreement and ensured that no errors occur during the review. All review authors critically reviewed, revised, and approved the subsequent and final version of the protocol.

**Conceptualization:** Xinmei Li, Yunyu Liang.

**Data curation:** Xinmei Lin, Qinyan Peng, Jiexiang Xiang.

**Formal analysis:** Xinmei Lin, Qinyan Peng, Xiaobin Yao, Jiexiang Xiang.

**Funding acquisition:** Xiaobin Yao.

**Resources:** Yuanping Wang.

**Software:** Yuanping Wang.

## Supplementary Material

Supplemental Digital Content
