# Supplementary material for: Acupuncture for hyperlipidemia: Protocol for a systematic review and meta-analysis
Source: Medicine (Baltimore). 2018 Dec 14;97(50):e13041. doi: 10.1097/MD.0000000000013041 (PMC6319778; doi:10.1097/MD.0000000000013041)
Supplement: Supplemental Digital Content [file medi-97-e13041-s001.docx]

**Appendix A.**

***Search strategy used in PubMed database***

#1 Hyperlipidemia

#2 Acupuncture

#3 Randomized controlled trial OR clinical study OR Clin-ical Trial OR Controlled study OR Controlled Trial OR Random*Control* study OR random* Control* Trial

#1 AND #2 AND #3
